# Health Care in Danger: Deliberate Attacks on Health Care during Armed Conflict

**DOI:** 10.1371/journal.pmed.1001668

**Published:** 2014-06-24

**Authors:** The PLOS Medicine Editors

## Abstract

The *PLOS Medicine* editors discuss the escalating problem of deliberate attacks on health care during armed conflict and support the importance of research on practical approaches to preventing such attacks, as well as studies that more broadly evaluate interventions to improve health care in conflict settings.

*Please see later in the article for the Editors' Summary*

Since 2001, June 20^th^ has been the day when the world considers the plight of refugees and internally displaced people by commemorating World Refugee Day [1]. This year's theme is a continuation of the “1” campaign, in which the world is asked to take 1 minute to consider the situation for a family forced to flee, many of whom may have just 1 minute to get ready [2]. Keeping with the time theme, the UN High Commissioner for Refugees (UNHCR) estimates that world-wide, one person is forced to flee to become a refugee or internally displaced person every 4.1 seconds [3].

One of the main drivers of displacement is armed conflict, which is the disruptive force responsible for most of the world's 45.2 million displaced people [3]. Providing health care to displaced people is challenging, even to those in relatively stable settings, such as camps. For example, earlier this month, *PLOS Medicine* published an article by Joshua Mendelsohn from the London School of Hygiene & Tropical Medicine and colleagues from the UNHCR calling for equity in antiretroviral therapy provision for refugees and internally displaced people with HIV. The authors focused on stable settings and proposed several recommendations mostly targeted at host countries [4]. Previously, *PLOS Medicine* published an article from Unni Karunakara from Médecines Sans Frontières and Frances Stevenson from HelpAge International highlighting the particular challenge of meeting the health needs of older people caught in conflict and other emergency settings [5].

The devastating effects of armed conflict on the health of populations is in no doubt, with both the direct effects of violence and the indirect effects, such as disruption to health services, having a huge toll on mortality and morbidity. For example, a study by Amy Hagopian and colleagues published in *PLOS Medicine* last year showed that, between 2003 and 2011, the majority of deaths in Iraq during the war and occupation were caused by the direct effects of violence and a third were due to indirect effects of health system disruption, resulting in a total of approximately half a million deaths attributable to the conflict [6].

To further add to the destruction and chaos of conflict, the past few years have brought mounting concern over the deliberate attacks on health care facilities and health workers, perpetrated to cause maximum damage to the health of populations. In 2011, the International Committee of the Red Cross (ICRC) published a landmark report that documented attacks on health care in 16 countries affected by conflict [7]. As the ICRC says: “Statistics represent only the tip of the iceberg: they do not capture the compounded cost of violence–health-care staff leaving their posts, hospitals running out of supplies, and vaccination campaigns coming to a halt” [7]. These knock-on effects of attacks dramatically limit access to health care for entire communities. Furthermore, such attacks are an insult to the Geneva Conventions, and the international community has responded with several initiatives and activities. For example, the ICRC launched the Health Care in Danger campaign, with the slogan “Violence against health care must end” [8]. And several organizations worldwide have recently joined forces to form the Safeguarding Health in Conflict Coalition, with the aim of promoting respect for international humanitarian and human rights laws for the safety of health facilities, health workers, ambulances, and patients during conflict [9].

Furthermore, in 2012, the World Health Assembly adopted a resolution (WHA 65.20) calling for the World Health Organization to improve reporting of, and data collection on, attacks on health care in conflict settings [10]. Then last November, an international conference in Bellagio, Italy on the protection of health workers, patients, and facilities in times of violence issued a call for action (targeted particularly at states and UN agencies but also at health professional organizations) to advance the security of health, particularly in situations of armed conflict and internal disturbances [11].

With such concerted activity attempting to tackle the egregious acts of attacks on health care, it is disappointing to note the distinct lack of progress in reducing the number of such attacks. A report by Human Rights Watch and the Safeguarding Health in Conflict Coalition, released to coincide with last month's World Health Assembly, catalogued recent examples of attacks on health workers and facilities [12]. The report makes depressing reading and provides explicit examples from 18 countries of attacks on health care, some better known than others. For example, in September 2013 the UN-mandated Independent International Commission on the Syrian Arab Republic stated that Syrian health workers and facilities have been deliberately and systematically targeted [12]. And the report states that since December 2013, South Sudan's conflict has led to widespread attacks on civilians, including in hospitals, and massive destruction of dozens of hospitals and clinics [10]. The report notes that the level of attacks has escalated recently and calls on the global community to recognize attacks targeted against health care as a critical human rights issue [12]. The report also adds to the Bellagio call for action and stresses that more action is urgently needed, including expanding and coordinating research on attacks and on the interference with health care, through in-depth qualitative studies [12].

*PLOS Medicine* supports the importance of research on practical approaches to prevent such attacks, as well as studies that evaluate interventions to improve health care in conflict settings more broadly. Such research is difficult and fraught with “real world” factors, but, as a recent article published in *PLOS Medicine* argues, disaster health interventions and decision-making can benefit from an evidence-based approach [13]. In this article, Martin Gerdin and colleagues from the initiative Evidence Aid argued that health care decision-making in disaster preparedness and response needs to move towards a reliable and robust evidence base for all interventions being considered in disaster risk reduction, planning, response, and recovery [13].

Deliberate attacks on patients, hospitals, and clinics are atrocious acts. While of course improved data collection on the number and nature of the attacks is important, practical action is also necessary to help improve the health outcomes of people terrorised, harmed, and displaced by such attacks. The *PLOS Medicine* editors welcome the research, debate, and discussion on how such practical measures can be implemented. Let's hope that next year's World Refugee Day will have more positive news.
